# A Facile One-Pot Preparation and Catalytic Application of Tunable Silica-Coated Aqueous Gold Nanoparticles

**DOI:** 10.3390/molecules30061355

**Published:** 2025-03-18

**Authors:** Elijah Cook, Kelly Moran, Qiaxian R. Johnson, Asmaa Lakhal, Bhanu P. S. Chauhan

**Affiliations:** Engineered Nanomaterials Laboratory, Department of Chemistry, William Paterson University of New Jersey, 300 Pompton Road, Wayne, NJ 07470, USA; elijahcook@g.ucla.edu (E.C.); morank18@student.wpunj.edu (K.M.); johnsonq6@wpunj.edu (Q.R.J.); asmaalakhal60@gmail.com (A.L.)

**Keywords:** trimethoxysilylpropyl-(polyethylenimine), siloxane-substituted polyethylenimine, morphology, nucleation and growth, aqueous, siloxane coating, self-assembled particles, size-dependent applications, catalytic reduction

## Abstract

It is known that designer polymers can be used for the synthesis and stabilization of metallic nanoparticle systems, providing new, tailorable properties. In this work, we demonstrate the trifold utility of a designer polymer, trimethoxysilylpropyl-(polyethylenimine) (TMSP-PEI), providing reduction, stabilization, and protection in a single step. Our facile and unique synthesis affords gold nanoparticles with varying sizes and morphologies in a range of solvents without the need for additional reducing agents. The use of this substituted polymer was manipulated in terms of the metal-to-ligand ratio to induce changes in the nanoparticle nucleation and growth. Upon further experimental analysis, it was discovered that adjustments to not only the metal–ligand ratio but also the solvent environment produced nanoparticles with different shape and size distributions. In addition, the synthesized gold nanoparticles were investigated for their catalytic ability to reduce Eosin Y in the presence of sodium borohydride without degradation.

## 1. Introduction

Polymeric materials play a critical role in the isolation and stabilization of metal nanoparticles [1,2,3,4,5,6,7].The synthesis of polymer-coated metal nanoparticles (NPs) has brought about many opportunities for their applications in catalysis as well as biological systems [8,9,10,11].The water-soluble polyelectrolyte polyethylenimine (PEI) has been extensively studied as a reducing and stabilizing agent for metal core nanoparticles [12,13,14,15,16,17,18,19,20,21,22,23,24]. Polyelectrolytes can provide stabilization through both electrostatic and steric methods, called electrosteric stabilization [25,26,27]. Through functionalization of PEI, the properties and sizes of the resulting nanoparticles can be tailored for specific applications [28,29,30,31]. Previous work has shown that aminosilane compounds are also capable of reduction and stabilization in various metal systems [32,33,34]. It is highly plausible that the modification of polyethylenimine with a siloxane-containing molecule will result in nanoparticles with new or enhanced properties.

**Figure 1:**
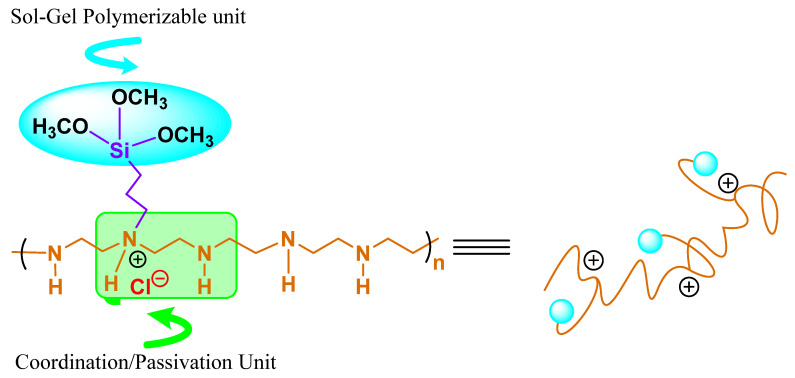
Structure of the TMSP-PEI polymer and its cartoon depiction.

Trimethoxysilylpropyl-(polyethylenimine) (TMSP-PEI) is a siloxane-modified PEI which can provide adjustable control over nanoparticle agglomeration and passivation [34]. The structure is depicted in Figure 1. The particle size, shape, charge, and solubility can be controlled via the stabilizing agent utilized [1]. By introducing a siloxane moiety, new routes for surface functionalization arise, as well as the possibility for gelation through the sol–gel process.

To exploit the multifaceted efficacy of TMSP-PEI, we investigated the application of this polymer as a reducing and stabilizing agent for gold nanoparticles in one pot and in various solvents. It was found that by adjusting the metal-to-ligand (TMSP-PEI) ratio as well as the solvent system, the nanoparticle morphology and size could be tailored. The catalytic performance of the nanoparticles was also examined by applying the TMSP-PEI Au NPs to catalyze the reduction of Eosin Y. This process can be carried out and monitored with UV–vis spectroscopy, providing a visual illustration of the catalytic ability.

## 2. Results and Discussion

**Figure 2:**
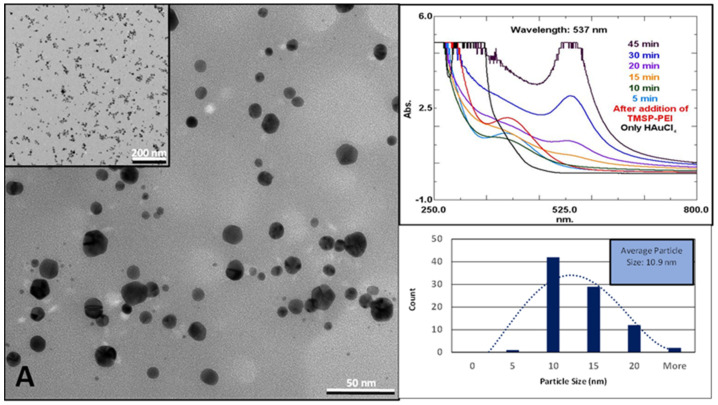

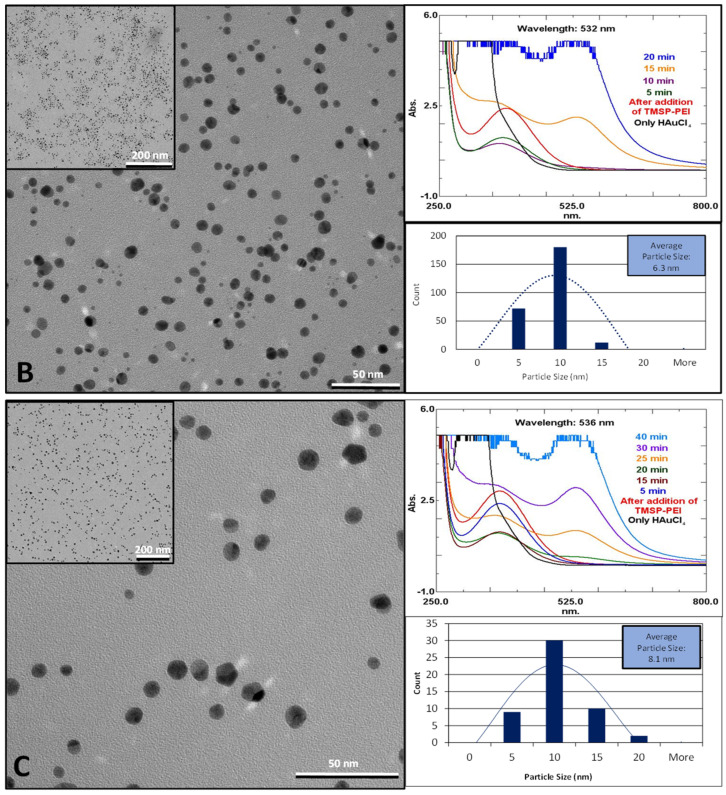
TEM analysis (50 nm, inset at 500 nm), UV–vis absorption spectra, and particle size analysis (PSA) of synthesized gold nanoparticles in isopropanol: (**A**) 1.6 equivalents of TMSP-PEI. (**B**) 3.5 equivalents of TMSP-PEI. (**C**) 14 equivalents of TMSP-PEI.

The initial synthesis of the nanoparticles showed that an increase in the alkoxysilane-substituted polymer impacted the particle size and shape, as evidenced by UV–vis and TEM analysis (Figure 2A–C). With 1.6 equivalents of the TMSP-PEI polymer, the particles were much larger and produced a mixture of morphologies. This dark pink solution had nanoparticles that appeared to be nucleated within the polymer system. The dark purple–red 3.5 equivalents reaction mixture displayed a more consistent morphology and smaller size regime. The average size of the nanoparticles (NPs) was slightly larger, with a greater size distribution, when 14 equivalents of the TMSP-PEI polymer were used. The larger particles were more irregular, with a percentage having truncated morphologies, while the smaller particles were spherical. The solution color of all synthesis ratios was dark purple/pink.

**Scheme 1:**
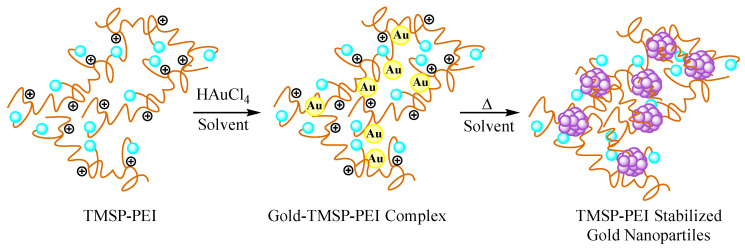
Schematic representation of the synthetic route to produce TMSP-PEI-stabilized gold nanoparticles.

A complex was observed via UV–vis analysis between the gold chloride trihydrate and the TMSP-PEI polymer. The gold chloride trihydrate showed a saturated absorption band at 320 nm. Immediately upon the addition of TMSP-PEI to the solution, the color turned from a bright yellow to a yellow–orange, and absorbance spectra showed what appeared to be a complexation between the metal salt and the substituted polymer at 380 nm. As the reaction progressed, the complexation peak decreased, and the reaction color became more red. When the solution turned peach-colored, the absorbance of the complexed nanoparticles equilibrated, and this where the first traditional absorbance was seen at ~530 nm. After this peak was observed, the area around 380 nm also increased as the reaction continued and saturated. A plausible mechanism for the reduction process is the bidentate complexation of gold salt to the TMSP-PEI polymer via adjacent nitrogen atoms on the polymer. This TMSP-PEI–gold complex under heating conditions led to the decomposition of the complex to Au^0^ particles. In situ stabilization of these particles via amino polymer using the same process led to the formation of stable gold nanoparticles, stabilized by amino ligands. It is possible that this 380 nm peak is representative of very small gold nanoparticles and/or complexes that “seed” the growth of bigger nanoparticles. Under the moderate reduction conditions provided by TMSP-PEI, the formation of Au complex clusters to initiate nucleation is likely [35]. This may explain why some of the ratios were unsuccessful under certain conditions. If there was not enough of these smaller particles/complex, the reaction could not be driven to completion due to sequestration. A schematic representation of the synthesis is provided in Scheme 1.

**Figure 3:**
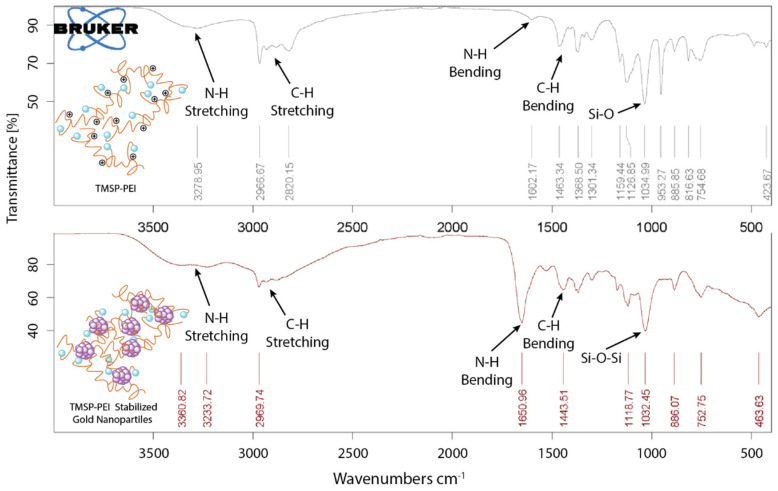
FTIR analysis of dried TMSP-PEI (**top**) and dried TMSP-PEI Au NPs at a 1:3.5 ratio (**bottom**).

In order to clearly identify and characterize the expected chemical changes in the nanoparticle synthesis, dried samples of TMSP-PEI and TMSP-PEI-conjugated gold nanoparticles were analyzed by FTIR spectroscopy (Figure 3). Ligand–metal coordination of the -NH_2_ of PEI with gold was verified via changes observed at ~1602–1650 cm^−1^ and ~3200–3400 cm^−1^, which are characteristic of N-H bending and stretching, respectively. A broadening of the sharp peak at ~1000 cm^−1^ was observed, which can be attributed to the formation of siloxane bonds. This is consistent with the branched siloxane formation that was expected with the coating observed in the TEM micrographs. Furthermore, the changes observed in terms of the C-H stretching at ~2800–3000 cm^−1^ as well as CH_2_ and CH_3_ bending modes at ~1300–1500 cm^−1^ were consistent with metal–PEI coordination and also with Si-O-Si bond formation [36,37].

**Figure 4:**
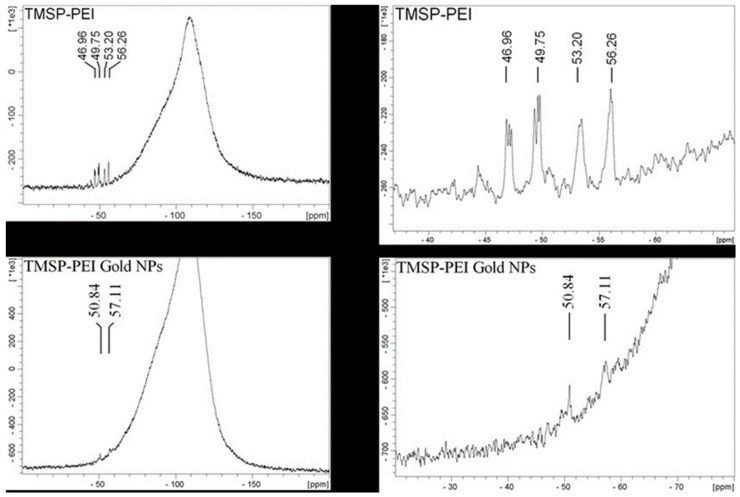
Liquid-state ^29^Si-NMR analysis of a small-scale synthesis of gold nanoparticles with TMSP-PEI. TMSP-PEI (3.5 equivalents in 2 mL of CD_3_OD) analysis at top. Gold chloride trihydrate (1 equivalent) was added, and the reaction was heated at 55 °C in an NMR tube, and the resultant nanoparticles were analyzed (bottom).

A small-scale synthesis of TMSP-PEI conjugated gold NPs was conducted in an NMR tube directly to follow the changes in the Si-O bond. TMSP-PEI (3.5 eq in 2 mL of CD_3_OD) was analyzed first and displayed peaks at −46.9, −49.7, −53.2, and −56.2 ppm in the ^29^Si-NMR (Figure 4). To this solution, 1 equivalent of gold chloride trihydrate was added, and the solution was heated to increase the reduction rate of gold chloride to gold nanoparticles. The heating of the reaction mixture also increased the hydrolytic condensation of Si–alkoxy bonds. The resulting ^29^Si-NMR of the synthesized TMSP-PEI-stabilized gold NPs displayed weak peaks at −50.8 and −57.1 ppm. These two peaks can be attributed to residual Si–alkoxy bonds, specifically T1 and T2 substructures. The hydrolytic condensation of T1 and T2 branching groups led to the formation of fully condensed Si-O-Si structures, causing the peak to merge into the glass peak of the NMR tube. The shift in the peaks during the reaction suggests the formation of Si-O-Si bonds or silica coating.

**Figure 5:**
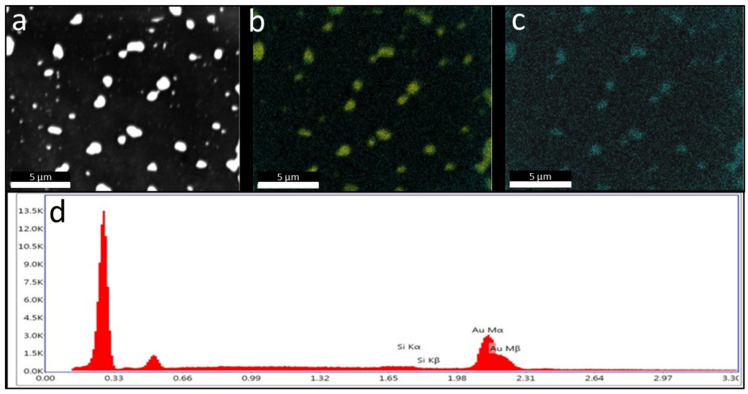
SEM and EDS analysis of TMSP-PEI-conjugated gold NPs. The solution (1:3.5 ratio, H_2_O) was dried, redispersed in H_2_O, drop-casted onto carbon tape, allowed to dry, and coated with carbon. EDS was performed at 5 kV, 5 µm, and with a probe current of 40 Å. (**a**) Image of area analyzed via EDS. (**b**) Elemental overlay of EDS: Au is yellow, Si is blue, and carbon tape background is black. (**c**) Silicon only elemental overlay. (**d**) EDS spectrum of the TMSP-PEI-conjugated gold nanoparticles.

The samples were dried and subjected to SEM and EDS analysis to elucidate the structural features as well as the composition of the TMSP-PEI-stabilized gold nanoparticles. The bright areas of the imaged area correlated with the gold/silicon overlay shown in Figure 5. The highly dense areas of silicon matched both overlays, indicating not only successful synthesis of the gold NPs but also the presence of a silica coating on the nanoparticles. It is noted that there were trace amounts of silica in the background. However, this was attributed to excess TMSP-PEI in the solution as well as the carbon tape used. In addition, the EDS spectrum detected carbon, gold, and nitrogen signals, confirming the successful synthesis of the gold nanoparticles.

While there was some siloxane coating around the nanoparticles, increasing the ratio of the TMSP-PEI polymer did not increase the coating thickness, as was expected. A possible explanation may be the low percentage of trimethoxysilypropyl substitution on the PEI, which, at the ratios analyzed, was appropriate and sufficient to reduce, stabilize, and coat the nanoparticles but was too low to cause a change in the shell thickness. With this ligand system, the nanoparticles were found to be stable for several weeks if stored at ambient conditions. After several weeks, gradual precipitation was observed.

**Scheme 2:**
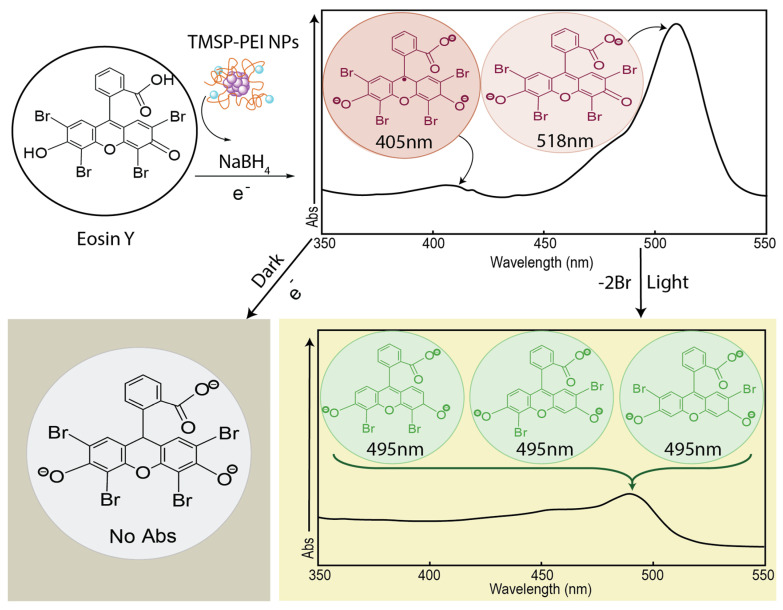
Reduction of Eosin Y catalyzed by TMSP-PEI gold nanoparticles. The spectra above were replotted/redesigned for schematic clarity from the original UV–vis spectra shown in Figure 6; 1:14 ratio reaction in dark and 1:3.5 ratio under natural light.

To illustrate the utility of the TMSP-PEI gold nanoparticles, they were applied as a catalyst for the reduction of common dyes such as Eosin Y [38,39,40]. There are two possible pathways for the reduction of Eosin Y: one-electron reduction and two-electron reduction [41,42,43,44,45]. In an aqueous solution, Eosin Y in the presence of sodium borohydride forms an EY^2−^ di-anion with a maximum absorbance at 510 nm. The most probable cause for this change is the action of sodium borohydride to produce the di-anion from the acidic carboxylic and phenolic protons. Sodium borohydride can reduce EY^2−^ to form EY^4−^, which is colorless and shows only a slight absorption in the UV range [41,42,43,44,45]. This traditional reduction of Eosin Y with sodium borohydride follows a two-electron reduction. The addition of gold nanoparticles to this system resulted in a one-electron reduction process. In the presence of the gold nanoparticles, the EY^2−^ was reduced through the transfer of a single electron to form the EY^3−^ radical, which showed a maximum absorbance at 405 nm. If the reaction was carried out in the dark, this unstable EY^3−^ radical was further reduced to EY^4−^. However, in the presence of natural light, the EY^3−^ radical underwent a photochemical fragmentation, resulting in the loss of two bromine atoms and the formation of a new green dye with an absorbance centered at 495 nm [41,42,43,44,45]. If the reaction was continued under illumination from a light, the green dye molecules were further fragmented to other colorless species. The overall reduction pathways for Eosin Y are depicted in Scheme 2.

TMSP-PEI nanoparticles synthesized in ratios of 1:1.6, 1:3.5, and 1:14 in water were used for the reduction of Eosin Y. The concentration of the synthesized nanoparticles was determined following the calculations carried out by Kedem and Langer [46]. This data are available in the supporting information (Table S1 Supplementary Materials).

**Figure 6:**
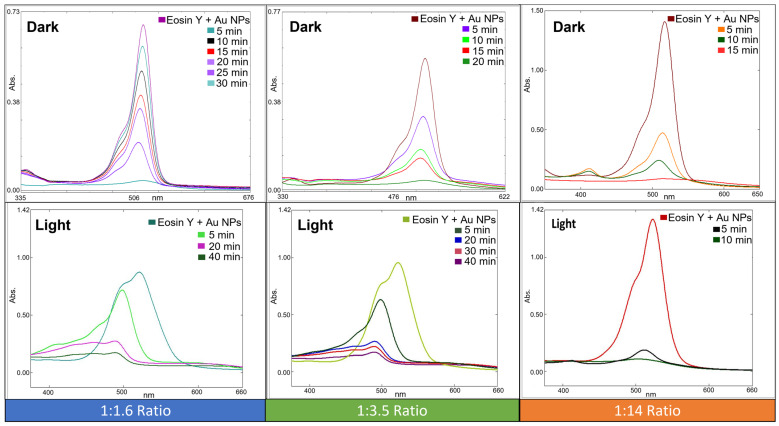
UV–vis analysis of Eosin Y reduction catalyzed by TMSP-PEI Au nanoparticles at different ratios and time intervals.

Two sets of reactions were carried out for each ratio: one in the dark and one under illumination with a blue LED light. The reactions were monitored using UV–vis spectroscopy, shown in Figure 6, until completion of the reaction. The nanoparticles all showed the successful reduction of Eosin Y at varying rates. Interestingly, the particle’s catalytic performance increased with higher ratios of TMSP-PEI ligand for both the light and dark reactions.

Stabilizing molecules coating nanoparticles are known to affect their catalytic performance by reducing the amount of free active sites on the particle’s surface [46,47]. For example, increasing the chain length for large polymer ligands decreases the packing density due to steric interactions, leading to an increase in catalytic performance [47]. Nanoparticles synthesized with different ratios of TMSP-PEI may have different packing densities, leading to differences in the diffusion of reactant molecules to the surface. A higher packing density with a more well-ordered structure can cause a larger diffusion barrier, restricting the access of small molecules to absorb onto active sites [47].

The reactions for all ratios under blue LED light had an increased rate compared to those carried out in the dark. This could have been due to the rapid depletion of the EY^3−^ radical through photochemical fragmentation, driving the reaction to completion with an increased rate. The green dye molecules can then be further reduced to a colorless species. The absorption peak for the nanoparticles following the reaction was slightly redshifted for all ratios. This could have been due to ligand removal during the reaction.

## 3. Materials and Methods

### 3.1. Materials

All reagents were used from the manufacturer as received without further purification. The polymer, TMSP-PEI, with a 20% substitution of NH_2_ units, was purchased from Gelest as a 50% solution in isopropanol (IPA). The approximate molecular weight of the TMSP-PEI was 1500–1800 g/mol, as stated by the manufacturer. Gold (III) chloride trihydrate, purchased from Thermo Fisher Scientific, was used as the gold precursor. Eosin Y (Tetrabromofluorescein), >95%, was purchased from TCI Chemicals. Sodium borohydride (98%) was acquired from MG Scientific and used as a reducing agent for the reduction of Eosin Y. UV–visible (UV–vis) absorption spectra were recorded on a Shimadzu UV-2550 spectrophotometer in 1 cm path-length quartz cuvettes to monitor the reaction. Transmission electron microscopy (TEM) micrographs to assess the morphology and size were acquired with a Hitachi HT7700 device with an accelerating voltage of 100 kV. All samples were prepared by drop-casting a single drop of the final reaction mixture onto a formvar-/carbon-coated copper grid and air-dried. The Image J program was used for all manual particle size analyses (PSA). A Hitachi SU1510 scanning electron microscope (SEM) and energy-dispersive X-ray spectroscopy (EDS) analysis were used to assess and verify the elemental composition of the gold nanoparticles. Fourier transform infrared spectra (FTIR) were recorded by a Bruker Vertex 70 FTIR operated in the transmittance mode to confirm the presence of characteristic chemical absorption peaks on solutions that were vacuum-dried. Nuclear magnetic resonance (NMR) analysis was conducted on a Bruker 400 Ultrashield device, which provided in situ experimental information on the gold nanoparticle synthesis.

### 3.2. Synthesis of Silica-Coated Gold Nanoparticles

In a standardized procedure, 0.1 mmol (0.0393 g) of gold chloride trihydrate was dissolved in 25 mL of IPA and placed in a pre-heated oil bath at 70 °C. A total of 0.16 mmol (0.0824 g) of stabilizing polymer solution, TMSP-PEI (20% substitution of NH_2_ units, as a 50% solution in isopropanol), was diluted in 25 mL of IPA. At this juncture, the diluted TMSP-PEI solution was added to the gold chloride solution and held at 70 °C. This reaction mixture was monitored using UV–vis spectroscopy. The reaction was assumed to be complete after 6 h, as the UV–vis spectra did not show any increase in absorbance after this time.

This procedure was followed to prepare the gold nanoparticles with various solvents and concentrations. Table 1 describes the solvent system, the temperature at which the reactions were carried out, the reaction times necessary to complete the reduction process, and the different molar ratios used.

The molar ratios for each synthesis were determined as follows. From the molecular weight of the TMSP-PEI monomer, 0.1 mmol was determined to be 0.0293 g. Using the density of the TMSP-PEI polymer, 1.055 g/mL, this value was converted to a volume of 0.0277 mL. As the TMSP-PEI polymer solution was 50% IPA, the volume was doubled to determine the amount of solution needed for 0.1 mmol of polymer (55.4 μL or 0.0515 g). Then, each ratio provided in the table below gave the approximation of the molar ratios of gold chloride to TMSP-PEI polymer used in the syntheses.

### 3.3. Experimental Procedure for the Catalytic Reduction of Eosin Y

#### 3.3.1. General Experimental Setup for the Investigation of the Catalytic Reduction of Eosin Y in the Dark

In all the reduction experiments, Eosin Y solution was freshly prepared. In a standardized procedure for the reduction of Eosin Y, a 1 mM stock solution of Eosin Y was prepared by dissolving 0.064 g of Eosin Y in 100 mL of deionized water. Separately, in a 1 cm path quartz cuvette containing 3 mL of deionized water, 31.5 μL of TMSP-PEI gold nanoparticles, as prepared, and 100 μL of the Eosin Y stock solution were mixed. To this mixture, 50 μL of freshly prepared 0.5 M sodium borohydride solution in water was added at room temperature. The cuvette was capped and shaken to mix the ingredients and start the reduction process. At this point, the cuvette was placed in the UV–vis spectrometer, which was already baselined using deionized water. The instrument was set to continually monitor the reaction progress via measuring the absorbance from 800 to 200 nm until the Eosin Y^2−^ peak was no longer present and the reaction was complete.

#### 3.3.2. General Experimental Setup for the Investigation of the Catalytic Reduction of Eosin Y in the Light

In a standardized procedure for the reduction of Eosin Y, a 1 mM stock solution of Eosin Y was prepared by dissolving 0.064 g of Eosin Y in 100 mL of deionized water. Separately, a 1 cm path quartz cuvette containing 3 mL of distilled water was prepared. To this cuvette, 100 μL of the 1 mM Eosin Y stock solution and 31.5 μL of the TMSP-PEI gold nanoparticles, as prepared, were mixed. At this point, a strip of generic blue LED lights was coiled around the cuvette and turned on. To the cuvette mixture, 50 μL of freshly prepared 0.5 M sodium borohydride solution in water was added at room temperature. The cuvette was capped and shaken to mix the ingredients and start the reduction process. The cuvette was then periodically placed into the UV–vis spectrometer every 5 min. The instrument was set to monitor the reaction progress via measuring the absorbance from 800 to 200 nm. Following each UV–vis measurement, the cuvette was returned to the LED lights until the next measurement. This process was continued until the reaction was complete.

## 4. Conclusions

The polymer TMSP-PEI was used to successfully reduce and stabilize metal nanoparticles with differing ratios in a variety of aqueous solvent systems. The resulting nanoparticle size and shape could be tailored by varying the metal salt-to-polymer concentration. Initially, during the synthesis, the gold chloride formed a complex with the polymer before the growth of the nanoparticles could take place. This complex, identified by its absorbance in the UV–vis analysis at 380 nm, was necessary for the reaction to be pushed to completion. These nanoparticles showed good catalytic ability for the reduction of Eosin Y without degradation. The tunability of this approach offers an adaptable method to synthesize nanoparticles particularly suited for various intended applications, such as bio-imaging, drug delivery systems, and other various bio-medical applications. Future work will show the application of this polymer system for the synthesis and utilization of different metal nanoparticles such as silver, platinum, palladium, and zinc.

## Figures and Tables

**Table 1:** Experimental conditions for gold nanoparticle synthesis in different solvents.

Solvent (25 mL), Reaction Time: 6 h			
Approximate Gold Chloride-to-TMSP-PEI Ratio	IPA (70 °C)	Methanol (55 °C)	Water (60 °C)
	Molar Amounts	Molar Amounts	Molar Amounts
	[Gram Amounts]	[Gram Amounts]	[Gram Amounts]
1:1.6	0.1 mmol:0.16 mmol	0.1 mmol:0.16 mmol	0.1 mmol:0.16 mmol
	[0.0393 g:0.0824 g]	[0.0393 g:0.0824 g]	[0.0393 g:0.0824 g]
1:3.5	0.1 mmol:0.35 mmol	0.1 mmol:0.35 mmol	0.1 mmol:0.35 mmol
	[0.0393 g:0.180 g]	[0.0393 g:0.180 g]	[0.0393 g:0.1800 g]
1:14	0.1 mmol:1.4 mmol	0.1 mmol:1.4 mmol	0.1 mmol:1.4 mmol
	[0.0393 g:0.721 g]	[0.0393 g:0.721 g]	[0.0393 g:0.721 g]

## Data Availability

The data presented in this study are available on request from the corresponding author.

## Supplementary Materials

The following supporting information can be downloaded at: https://www.mdpi.com/article/10.3390/molecules30061355/s1, Table S1: Nanoparticle size analysis of TMSP-PEI nanoparticles in ratios of 1–1.6, 1–3.5, and 1–14. and nanoparticle solution concentration calculations.

## Abbreviations

The following abbreviations are used in this manuscript:

NPs	Nanoparticles
Au NPs	Gold nanoparticles
PEI	Polyethylenimime
TMSP-PEI	Trimethoxysilylpropyl-(polyethylenimime)
Eosin Y	Tetrabormofluorescein
IPA	Isopropanol
