# Supplementary material for: A Facile One-Pot Preparation and Catalytic Application of Tunable Silica-Coated Aqueous Gold Nanoparticles
Source: Molecules. 2025 Mar 18;30(6):1355. doi: 10.3390/molecules30061355 (PMC11946824; doi:10.3390/molecules30061355)
Supplement: Supplementary file 1 [file molecules-30-01355-s001.zip › molecules-3525014-supplementary.pdf]

"Facile One-Pot Preparation and Catalytic Application of Tunable Silica-Coated Aqueous Gold Nanoparticles"

*Elijah Cook, Kelly Moran, Qiaxian R. Johnson, Asmaa Lakhal, and Bhanu P. S. Chauhan**

Engineered Nanomaterials Laboratory, Department of Chemistry, William Paterson University, 300 Pompton Road, Wayne, NJ 07470-2103, USA.

Supporting Information

TMSP-PEI Au NP Characterization:

Nanoparticle size analysis was conducted using TEM images with the NIH program ImageJ. The resulting data is shown below.

Table S1. Nanoparticle size analysis of TMSP-PEI nanoparticles in ratios 1-1.6, 1-3.5, and 1-14.

1-1.6 Ratio		1-3.5 Ratio		1-14 Ratio	
#	Length (nm)	#	Length (nm)	#	Length (nm)
**1**	11.267	1	3.91	1	5.591
**2**	10.267	2	3.963	2	7.103
**3**	11.846	3	3.754	3	9.066
**4**	12.593	4	4.848	4	8.611
**5**	10.95	5	5.288	5	10.817
**6**	9.587	6	6.62	6	7.443
**7**	10.961	7	4.639	7	7.762
**8**	16.624	8	4.667	8	6.957
**9**	12.935	9	5.67	9	5.592
**10**	6.425	10	5.575	10	9.729
**11**	10.438	11	6.665	11	9.136
**12**	10.553	12	4.745	12	7.739
**13**	12.596	13	5.71	13	8.451
**14**	8.139	14	5.256	14	6.986
**15**	10.746	15	7.941	15	9.313
**16**	9.539	16	7.54	16	7.994
**17**	11.477	17	7.705	17	10.439
**18**	11.425	18	5.269	18	9.809
**19**	11.275	19	4.291	19	8.298
**20**	4.756	20	5.885	20	9.161
**21**	9.323	21	5.604	21	8.101
**22**	9.049	22	6.021	22	7.634
**23**	11.437	23	7.236	23	8.364
**24**	8.445	24	4.689	24	6.838
**25**	12.638	25	3.91	25	7.793
**26**	11.28	26	5.944	26	8.576

**27**	11.662	27	5.505	27	8.236
**28**	9.797	28	7.121	28	10.025
**29**	11.597	29	5.545	29	8.282
**30**	15.531	30	4.518	30	6.073
**31**	10.688	31	4.973	31	7.56
**32**	10.325	32	6.662	32	10.035
**33**	11.848	33	7.654	33	10.219
**34**	11.912	34	5.532	34	7.576
**35**	12.138	35	5.441	35	6.756
**36**	9.186	36	4.905	36	6.341
**37**	12.862	37	4.873	37	7.883
**38**	11.004	38	6.877	38	8.359
**39**	8.614	39	6.877	39	7.06
**40**	4.809	40	6.451	40	9.725
**41**	10.226	41	5.831	41	7.385
**42**	12.72	42	6.562	42	6.438
**43**	8.885	43	5.315	43	5.239
**44**	9.776	44	5.206	44	10.744
**45**	10.212	45	6.434	45	10.035
**46**	9.785	46	6.727	46	9.48
**47**	12.932	47	7.295	47	10.859
**48**	13.337	48	6.622	48	5.61
**49**	8.617	49	6.383	49	9.638
**50**	5.237	50	5.43	50	11.801
**51**	7.326	51	4.605	51	13.822
**52**	7.376	52	6.889	52	12.071
**53**	12.485	53	8.175	53	8.781
**54**	10.401	54	8.404	54	9.038
**55**	7.384	55	5.866	55	9.378
**56**	9.326	56	5.562	56	7.646
**57**	12.128	57	5.572	57	8.849
**58**	3.329	58	7.808	58	9.179
**59**	7.558	59	6.341	59	5.061
**60**	10.913	60	9.232	60	9.943
**61**	8.089	61	5.914	61	4.231
**62**	9.934	62	7.083	62	11.705
**63**	13.918	63	6.717	63	10.4
**64**	14.708	64	7.816	64	11.674
**65**	9.102	65	5.396	65	8.418
**66**	12.258	66	6.252	66	11.9
**67**	15.561	67	6.239	67	7.711
**68**	10.867	68	7.956	68	8.657
**69**	12.286	69	8.818	69	10.963

**70**	9.149	70	7.721	70	9.765
**71**	12.126	71	7.265	71	8.813
**72**	12.943	72	7.758	72	9.313
**73**	8.952	73	6.382	73	4.117
**74**	16.638	74	9.216	74	13.052
**75**	11.298	75	8.131	75	6.574
**76**	13.463	76	8.596	76	11.453
**77**	6.439	77	7.455	77	9.231
**78**	6.864	78	9.677	78	3.813
**79**	9.854	79	6.914	79	8.915
**80**	9.805	80	6.586	80	5.263
**81**	12.369	81	6.93	81	10.337
**82**	9.369	82	9.296	82	3.536
**83**	12.995	83	6.788	83	3.779
**84**	10.783	84	10.341	84	9.536
**85**	10.846	85	9.197	85	7.81
**86**	13.305	86	5.285	86	4.882
**87**	11.278	87	7.026	87	7.854
**88**	13.389	88	7.113	88	6.209
**89**	10.563	89	7.375	89	9.669
**90**	8.731	90	5.229	90	4.53
**91**	12.586	91	8.394	91	8.127
**92**	13.723	92	8.322	92	6.429
**93**	11.259	93	6.093	93	3.689
**94**	8.508	94	6.433	94	10.788
**95**	8.141	95	6.411	95	9.762
**96**	16.708	96	6.932	96	5.129
**97**	10.491	97	5.649	97	3.419
**98**	10.809	98	7.067	98	11.862
**99**	12.258	99	7.975	99	8.81
**100**	12.451	100	5.595	100	6.667
**Average (nm):**	10.73313	Average (nm):	6.47881	Average (nm):	8.27222

The average diameter of the nanoparticles calculated with the program, ImageJ, and the initial mass of Gold (III) Chloride Trihydrate was used to determine the concentrations of the nanoparticle solutions. The calculations are provided below.

Mass of pure Au in reaction = (Mass of $HAuCl_4 \cdot 3H_2O$) · Mass fraction Au
$= 0.0393 \text{ g} \cdot 0.5 = 1.97 \times 10^{-2} \text{ g}$
Volume of Au added = Mass of Au added ÷ Density of Au
$= 1.97 \times 10^{-2} \text{ g} \div 19.32 \text{ g} \cdot \text{cm}^3 = 1.02 \times 10^{-3} \text{ cm}^3$
Volume of 1 Au NP = $\frac{4}{3} \cdot \pi \cdot r_{AuNP}^3 = \frac{4}{3} \cdot \pi \cdot (10.7 \text{ nm})^3 = 5{,}130 \text{ nm}^3$ (1-1.6 ratio)
$= \frac{4}{3} \cdot \pi \cdot (6.47 \text{ nm})^3 = 1{,}050 \text{ nm}^3$ (1-3.5 ratio)

$= \frac{4}{3} \cdot \pi \cdot (8.27\ nm)^3 = 2{,}230\ nm^3$ (1-14 ratio)

Number of NPs in solution = Volume of Au added ÷ Volume of 1 Au NP in cm^3

$= 1.02 \times 10^{-3}\ cm^3 \div 5.13 \times 10^{-18}\ cm^3 = 1.98 \times 10^{14}$ (1-1.6 ratio)

$= 1.02 \times 10^{-3}\ cm^3 \div 1.05 \times 10^{-18}\ cm^3 = 9.68 \times 10^{14}$ (1-3.5 ratio)

$= 1.02 \times 10^{-3}\ cm^3 \div 2.23 \times 10^{-18}\ cm^3 = 4.56 \times 10^{14}$ (1-14 ratio)

Concentration of stock Au NP solution = (Number of NPs in solution ÷ 6.022×10^{23} 1/mol) 0.1 L

$= (1.98 \times 10^{14} \div 6.022 \times 10^{23}\ 1/mol) \div 0.05\ L = 6.58 \times 10^{-9}\ M$ (1-1.6 ratio)

$= (9.68 \times 10^{14} \div 6.022 \times 10^{23}\ 1/mol) \div 0.05\ L = 3.22 \times 10^{-8}\ M$ (1-3.5 ratio)

$= (4.56 \times 10^{14} \div 6.022 \times 10^{23}\ 1/mol) \div 0.05\ L = 1.51 \times 10^{-8}\ M$ (1-14 ratio)
